# Mechanism of Ferroptosis and Its Relationships With Other Types of Programmed Cell Death: Insights for Potential Interventions After Intracerebral Hemorrhage

**DOI:** 10.3389/fnins.2020.589042

**Published:** 2020-11-13

**Authors:** Sheng-Yu Zhou, Guo-Zhen Cui, Xiu-Li Yan, Xu Wang, Yang Qu, Zhen-Ni Guo, Hang Jin

**Affiliations:** ^1^Department of Neurology, Stroke Center, The First Hospital of Jilin University, Changchun, China; ^2^Department of Hepatology, Cancer Center, The First Hospital of Jilin University, Changchun, China; ^3^Clinical Trial and Research Center for Stroke, Department of Neurology, The First Hospital of Jilin University, Changchun, China

**Keywords:** ferroptosis, intracerebral hemorrhage, iron, lipid peroxides, glutathione, programmed cell death

## Abstract

Intracerebral hemorrhage (ICH) is a fatal cerebrovascular disease with high morbidity and mortality, for which no effective therapies are currently available. Brain tissue damage caused by ICH is mediated by a newly identified form of non-apoptotic programmed cell death, called ferroptosis. Ferroptosis is characterized by the iron-induced accumulation of lipid reactive oxygen species (ROS), leading to intracellular oxidative stress. Lipid ROS cause damage to nucleic acids, proteins, and cell membranes, eventually resulting in ferroptosis. Numerous biological processes are involved in ferroptosis, including iron metabolism, lipid peroxidation, and glutathione biosynthesis; therefore, iron chelators, lipophilic antioxidants, and other specific inhibitors can suppress ferroptosis, suggesting that these modulators are beneficial for treating brain injury due to ICH. Accumulating evidence indicates that ferroptosis differs from other types of programmed cell death, such as necroptosis, apoptosis, oxytosis, and pyroptosis, in terms of ultrastructural characteristics, signaling pathways, and outcomes. Although several studies have emphasized the importance of ferroptosis due to ICH, the detailed mechanism underlying ferroptosis remains unclear. This review summarizes the available evidence on the mechanism underlying ferroptosis and its relationship with other types of cell death, with the aim to identify therapeutic targets and potential interventions for ICH.

## Introduction

Intracerebral hemorrhage (ICH) accounts for 10–15% of all stroke types, which is a catastrophic event associated with high mortality and morbidity rates (Van Asch et al., 2010). Edema in a bleeding brain after ICH contributes to secondary brain injury (SBI). Current ICH treatments include dehydration treatment, antihypertensive therapy, platelet transfusion, and surgery. However, these therapies have some limitations; for example, there is not sufficient evidence to support the safety of platelet transfusion, and dehydration treatment causes a rapid decrease in blood pressure in a specific area (Morotti and Goldstein, 2016). Moreover, the mechanism underlying SBI after ICH remains unclear. However, it is known that hemoglobin in a hematoma after ICH can cause lipid peroxidation and form hydroxyl radicals, which are highly neurotoxic, leading to damage to the membranes, DNA, and proteins (Xi et al., 2006; Cao and Dixon, 2016). As the key element of hemoglobin, iron also plays an important role in SBI. Iron toxicity is mainly based on the Fenton reaction. Through the Fenton reaction, iron forms a large number of hydroxyl radicals, which trigger and aggravate the oxidation of tissues, causing neuronal cell damage (Zhao et al., 2011b; Auriat et al., 2012; He et al., 2020). Some preclinical and clinical studies about ferroptosis in neurodegeneration and stroke indicate that iron released from the hematoma can lead to a newly identified form of programmed cell death, ferroptosis, which may greatly contribute to SBI after ICH (Zhang Z. et al., 2018; Djulbegovic and Uversky, 2019; Zhang et al., 2020). Ferroptosis was first defined in 2012 (Dixon et al., 2012); it refers to an oxidative and iron-dependent form of programmed cell death that differs from other known types of programmed cell death in terms of its morphological and biochemical features, including the ultrastructural characteristics, signaling pathways, and outcomes (Zille et al., 2017; Bobinger et al., 2018; Hirschhorn and Stockwell, 2019). Ferroptosis is associated with iron and amino acid metabolism together with lipid peroxidation, with the iron-dependent accumulation of lipid peroxidation being the key trigger (Dixon et al., 2014; Conrad et al., 2018; Fujii et al., 2019; Hirschhorn and Stockwell, 2019). In this process, inhibition of glutathione peroxidase 4 (GPX4) prevents the appropriate reduction of lipid peroxides, resulting in increased generation of reactive oxygen species (ROS), which in turn leads to oxidative lipid damage, protein aggregation, and ultimately, neuronal cell death (Gao et al., 2015; Bai et al., 2019).

Although the mechanism of ferroptosis is not entirely understood, the use of iron chelators, ferroxidase, and other mediators of ferroptosis may be effective as a potential therapeutic approach for ICH. Strategies to inhibit or delay the rate of iron accumulation, glutathione (GSH) depletion, and lipid peroxidation are currently being tested in trials (Gao et al., 2015; Wang et al., 2016; Conrad et al., 2018). Therefore, in this review, we present the available evidence on the mechanisms underlying ferroptosis to highlight candidate therapeutic targets and potential interventions for ICH. We further discuss the relationships between ferroptosis and other types of cell death. This overview will not only provide a better understanding of the clinical significance of ferroptosis but also promote the progress of research regarding novel aspects, such as the development of potential anti-ferroptotic strategies to prevent SBI after ICH.

## Mechanisms Underlying Ferroptosis Following ICH

### Iron Metabolism

Iron is essential for excessive lipid peroxidation, which leads to ferroptosis, particularly in ICH (Dixon et al., 2012). Therefore, iron uptake, utilization, export, and storage can affect nearly all ferroptosis processes. ICH occurs upon vessel rupture; thereafter, blood enters the surrounding parts of the brain, causing an intracerebral hematoma, which releases hemoglobin, heme, and iron into the surrounding tissue (Li et al., 2019). Hemoglobin, the most abundant protein in the blood, can act as a neurotoxin released from lysed red blood cells after hematoma formation (Xi et al., 2006). Two forms of iron are involved in this process: ferric (Fe^2+^) and ferrous (Fe^3+^) iron. Fe^2+^ can transfer electrons and has high solubility; thus, Fe^2+^-containing proteins serve as cofactors and catalysts in several types of oxidation–reduction reactions. In contrast, Fe^3+^ stores and transports iron and is, therefore, much more stable than the active Fe^2+^ (Dixon et al., 2012). Under physiological conditions, transferrin (Tf) recognizes Fe^3+^ and binds two Fe^3+^ molecules to form diferric Tf, which subsequently binds to the Tf receptor 1 (TfR1) with high-affinity to form the Tf-Fe^3+^-TfR1 complex on the surface of neurons. The Tf-Fe^3+^-TfR1 complex is then transported into cells via endocytosis and then into endosomes, where iron is released from the complex because of the acidic environment. Free Fe^2+^ is then reduced to Fe^3+^ and stored in the labile iron pool (LIP; Hentze et al., 2010). The Fe^2+^ in the LIP reacts with hydrogen peroxide, thereby forming hydroxyl radicals via the Fenton reaction (Gao et al., 2015; Feng et al., 2020; He et al., 2020). These highly toxic hydroxyl radicals attack the lipid membrane, DNA, and proteins to cause lipid ROS production, disrupting cellular function and leading to ferroptosis. Moreover, iron can be exported from the cells by ferroportin, which contributes to the reduction of the intracellular Fe^2+^ concentration. Therefore, ferroportin may serve as a potential therapeutic target for ICH. However, Li et al. (2019) showed that the hemoglobin content and hematoma volume gradually decrease after ICH, and they illustrated that the endogenous clearance system can be activated and eliminates the hematoma after ICH. Several studies have indicated that the decrease in hematoma volume might be related with erythrophagocytosis (Cao et al., 2016; Ni et al., 2016; Chang et al., 2020). Since intraparenchymal hematomas and red blood cells are the major source of free iron in the ICH brain, hematoma resolution and the clearance and phagocytosis of red blood cells might reduce iron-induced ferroptosis. Further studies are warranted to investigate these approaches to tackle iron-induced ferroptosis.

### Depletion of GSH

Glutathione biosynthesis is intricately linked to the regulation of ferroptosis. As a physiological defense under normal conditions, ROS can be maintained at a stable level via mitochondrial oxidative phosphorylation and antioxidant mechanisms. However, upon ICH, substantial amounts of ROS are produced, which cause cell damage, ultimately leading to ferroptosis. This event activates GSH in the brain as an antioxidant enzyme. Thus, the inhibition of GSH synthesis is predicted to induce ferroptosis. Intracellular glutamate is exchanged for cysteine, and system Xc- transports cysteine into neurons at a 1:1 ratio. Cysteine, glutamate, and glycine are used in GSH synthesis (Dixon et al., 2014; Gao et al., 2015), whereas erastin can inhibit the expression of system Xc-, causing GSH depletion and ultimately leading to ROS accumulation (Dixon et al., 2012). Therefore, the concentration and balance of these amino acids can impact ferroptosis. For example, high extracellular concentrations of glutamate and low intracellular concentrations of cysteine inhibit the function of system Xc- and induce ferroptosis. This may explain the toxic effects of glutamate following its accumulation at high levels in the nervous system.

### Lipid Peroxidation

Lipid peroxidation is the key process that directly activates ferroptosis. It involves three pathways: (i) the non-enzymatic pathway generates lipid ROS through the Fenton reaction using iron (Bai et al., 2019; He et al., 2020); (ii) the oxygenation and esterification of polyunsaturated fatty acids generate lipid peroxides (Yang et al., 2016; Doll and Conrad, 2017; Stockwell, 2018; Das, 2019); and (iii) iron catalyzes lipid auto-oxidation. Some studies have reported that exogenous arachidonic acid (AA) and AA-OOH-phosphatidylethanolamine (PE) enhance ferroptosis. The conversion of AA-OOH-PE to AA in cells requires lipoxygenase enzymes. AA-OOH-PE is then reduced to AA-OH-PE by GPX4. Ferroptosis occurs when the AA-OH-PE levels are sufficiently high to surpass the cellular threshold (Kagan et al., 2017). Under physiological conditions, toxic lipid peroxides are reduced to non-toxic lipid alcohols by GPX4 to protect cells against oxidative stress (Kagan et al., 2017; Zhang Z. et al., 2018; Vuckovic et al., 2020). However, substantial accumulation of lipid peroxides cannot be effectively eliminated by GPX4, and the excess lipid peroxide damages membrane integrity, leading to cell rupture and ferroptosis, which has been proven in proteomics studies (Forcina and Dixon, 2019; Fujii et al., 2019). Therefore, the processes that inhibit the reduction in lipid peroxide levels and promote their formation result in the accumulation of lipid peroxides and, ultimately, ferroptosis (Stoyanovsky et al., 2019). However, the lipid autoxidation in ICH is not completely clear and should be further examined (Qiu et al., 2020).

## Relationships Between Ferroptosis and Other Cell Death Pathways

Table 1 shows an overview and comparison of different neuronal cell death types: necroptosis, apoptosis, oxytosis, and pyroptosis. Each type, along with its characteristics and mechanisms, and their potential roles in brain damage after ICH, are discussed below, and are compared with the corresponding features of ferroptosis.

**TABLE 1:** Comparison of different neuronal cell death types.

Types of cell death	Activators	Mediators	Inhibitors	Outcome
Ferroptosis	Iron, extracellular glutamine	Fe(II), ROS	Iron chelators, ferroxidase, antioxidants	Necrosis via lipid ROS
Necroptosis	Inflammatory factors	NLRP1/3, MKLK	Necrostatin-1, caspase-8	Necrosis via MKLK
Apoptosis	Inflammatory factors	Caspase 3,6,7,8,9	Bcl-2,zVAD	Phagocytosis
Oxytosis	Glutamate	System Xc-	BID inhibitors	Necrosis via glutamate
Pyroptosis	Inflammatory factors	Caspase-1, gasdermin D	NLRP1 inflammasome inhibitors	Inflammatory necrosis

### Ferroptosis and Necroptosis

Numerous reports have suggested that ferroptosis is always accompanied by necroptosis. Necroptosis is activated during inflammation (Oberst, 2016). The activation of necroptosis relies on receptor-interacting kinase 1 (RIPK1), receptor-interacting protein kinase 3 (RIPK3), and mixed lineage kinase domain-like (MLKL; Dondelinger et al., 2014; Conrad et al., 2016). RIPK1 activates RIPK3 and thereby recruits MLKL at the cell membrane, which causes membrane rupture and eventually triggers necroptosis (Cook et al., 2014; Hildebrand et al., 2014). Some studies have demonstrated that the ultrastructure of neurons after ICH manifests as a morphotype, involving ferroptosis and necroptosis. Ferroptosis is characterized by the loss of plasma membrane integrity, disruption and swelling of organelles, and shrunken mitochondria, whereas necroptosis does not involve mitochondrial shrinkage, suggesting that shrunken mitochondria are a characteristic change in ferroptosis after ICH (Li et al., 2018). The major ultrastructural characteristics of hemin-induced neuron death are related to ferroptosis and not necroptosis. In contrast, molecular marker levels of both ferroptosis (Fe^3+^ iron, GSH, and GPX4) and necroptosis (MLKL and RIPK3) may increase after ICH; however, such studies regarding ICH are lacking (Roderick et al., 2014; Lin et al., 2016; Newton et al., 2016; Minagawa et al., 2020). NADPH might be a link between ferroptosis and necroptosis (Hou et al., 2019). When cells undergo necroptosis or ferroptosis, adjacent cells are more prone to another form of cell death. Moreover, necrostatin-1 was shown to exert its neuroprotective effects by inhibiting the RIPK1 and RIPK3 signal pathways of necroptosis and suppressing apoptosis and autophagy after ICH (Chang et al., 2014; Cook et al., 2014; Su et al., 2015), highlighting its relevance to necroptosis, apoptosis, and autophagy; however, this does not clarify the specific link between ferroptosis and necroptosis, which require further studies.

### Ferroptosis and Apoptosis

Apoptosis is one of the most well-studied forms of programmed cell death. It includes two major pathways: extrinsic and intrinsic pathways. The extrinsic pathway is triggered by cell surface receptors, such as tumor necrosis factor (TNF) receptors (Hasegawa et al., 2011; Fricker et al., 2018; Zhao et al., 2018). The TNF receptors can be activated by caspase-1, which is stimulated by the P2X7 receptor (Lee et al., 2016). Upon binding to the TNF receptor, the Fas-associated death domain protein will be recruited and will bind pro-caspase-8 molecules, activating caspase-8 and allowing apoptosis to occur (Micheau and Tschopp, 2003). The intrinsic pathway is activated by the mitochondrial outer membrane permeabilization (MOMP) and the B-cell lymphoma 2 (Bcl-2) family proteins. As a member of Bcl-2 family, Bax can promote apoptosis by its expression and activation (Haase et al., 2008). Furthermore, Zhang et al. showed that the increased Bax/Bcl-2 ratio can promote apoptosis (Zhang W. et al., 2018). Therefore, Bcl-2 can be considered as an inhibitor of apoptosis. The ultrastructural features of an apoptotic neural cell include chromatin condensation, nuclear shrinkage, and DNA fragmentation (Castagna et al., 2016; Li et al., 2018). Compared with other types of cell death, DNA fragmentation can be considered as the distinguishing characteristic of apoptosis. Therefore, the presence of DNA fragments indicates apoptosis, which can occur at a later ICH phase (Kanter et al., 2016). Ferroptosis is an iron-dependent form of programmed cell death, but it is non-apoptotic, non-necroptotic, and non-autophagic. Ferroptosis can also be inhibited by iron chelators and several novel small molecules, such as ferrostatin-1 and liproxstatin-1, but cannot be inhibited by specific inhibitors of apoptosis (e.g., zVAD; Thermozier et al., 2020) or necroptosis (e.g., necrostatin-1; Chang et al., 2014; Chen X. et al., 2019).

### Ferroptosis and Oxytosis

Oxytosis is induced by the glutamate-mediated inhibition of system Xc-, which leads to the depletion of GSH; it represents a distinct type of oxidative neuronal cell death after ICH (Landshamer et al., 2008; Grohm et al., 2010). Depletion of GSH causes excessive ROS production, which cannot be eliminated, leading to ROS accumulation in neurons and, ultimately, oxytosis (Tobaben et al., 2010; Reuther et al., 2014; Jelinek et al., 2016). Oxytosis is highly similar to ferroptosis; it has even been regarded as a component of ferroptosis (Lewerenz et al., 2018; Gupta et al., 2020). A previous study suggested that BID protein inhibitors can prevent erastin-induced ferroptosis (Neitemeier et al., 2017), and that inhibitors of ferroptosis, such as ferrostatin-1 and liproxstatin-1, can block glutamate-induced oxytosis. The study further showed that erastin-induced ferroptosis in neuronal cells is accompanied by mitochondrial transactivation of BID, loss of mitochondrial membrane potential, enhanced mitochondrial fragmentation, and reduced ATP levels. These signs of mitochondrial death are distinctive features of oxytosis (Lewerenz et al., 2018; Takashima et al., 2019; Nagase et al., 2020). However, because ferroptosis cannot be readily distinguished from oxytosis, the relationship between ferroptosis and oxytosis remains unclear and controversial. Therefore, further research is needed to clarify the differences between these two types of cell death.

### Ferroptosis and Pyroptosis

Pyroptosis is another type of neuronal cell death and is activated by gasdermin D (GSDMD) and caspase-1 or caspase-11 in mice (corresponding to caspase-1, caspase-4, and caspase-5, respectively, in humans), consequently upregulating cytokines [interleukin (IL)-1β, IL-18] and immune system activators (Ruehl and Broz, 2015; Chen K.W. et al., 2019). Upon activation, caspase-1, or caspase-11 (in mice) acts on GSDMD and generates the N-terminal domain and C-terminal domain, and the lipid-selective N-terminal domain acts on the cell membrane by combining with phosphatidylinositol of the lipid plasma membrane, which leads to cell lysis. Cytokines such as IL-1β and IL-18 are secreted through the damaged cell membrane, recruiting immune cells, and activating the immune system, and ultimately resulting in pyroptosis (Shi et al., 2015; Ding et al., 2016; Feng et al., 2018). Pyroptosis is characterized by nuclear condensation coupled with cell swelling and lipid membrane vacuole formation at the plasma membrane, which eventually ruptures without exhibiting DNA fragmentation (Li et al., 2018), making the process distinct from ferroptosis. The activation of caspase-1 relies on the accumulation of a type of protein complex known as the inflammasome. Nucleotide-binding oligomerization domain-like receptor 3 (NLRP3) protein binds to apoptosis-related speck-like protein through its pyridine domain and recruits pro-caspase-1 via CARD–CARD interaction, forming NLRP3 inflammasomes (Li et al., 2020). NLRP3 can also be activated by K^+^ efflux, which is indirectly induced by caspase-11. In addition to recruiting and activating caspase-1 via apoptosis-related speck-like protein, inflammasomes can also bind with caspase-8 to induce cell death. Some studies also found that other members of the gasdermin family (e.g., GSDMA, GSDMB, and GSDMC) are recruited to the plasma membrane to trigger membrane repair upon gasdermin D activation and act on membrane-disrupting cytotoxicity (Denes et al., 2015; Man and Kanneganti, 2015; Shi et al., 2015; Chen et al., 2016). Therefore, pyroptosis has attracted substantial attention as an ICH therapeutic target, and inhibitors of NLRP3 were shown to have therapeutic efficiency. The biochemical processes of ferroptosis are much simpler than those of pyroptosis.

## Potential Interventions Targeting Ferroptosis After ICH

The most promising anti-ICH drugs targeting ferroptosis are summarized in Table 2. Ferroptosis is mainly activated by Fe^2+^ and ROS; thus, iron chelators and inhibitors of lipid ROS generation can be regarded as candidate therapeutic targets for ICH. Here, we focus on the potential targets, including iron metabolism, GSH biosynthesis, and lipid ROS.

**TABLE 2:** Promising anti-ICH drugs that target ferroptosis.

Drugs	Target	Impact on ferroptosis	References
Deferoxamine (DFO)	Iron	Function as iron chelator, depletes iron and prevent iron-dependent lipid peroxidation	Hu et al., 2019
Deferiprone (DFP)	Iron	Function as iron chelator, depletes ferric iron and prevent iron-dependent lipid peroxidation	Garringer et al., 2016
Minocycline	Iron	Function as iron chelator, depletes iron and prevent iron-dependent lipid peroxidation	Zhao et al., 2011a; Chang et al., 2017; Fouda et al., 2017; Yang et al., 2019, 2020
Nitrilotriacetic Acid (NTA)	Iron	Function as iron chelator, depletes iron and prevent iron-dependent lipid peroxidation	Kose et al., 2019
Ethylenediaminetetraacetic Acid (EDTA)	Iron	Function as iron chelator, depletes iron and prevent iron-dependent lipid peroxidation	Kose et al., 2019
Clioquinol (CQ)	Iron	Function as iron chelator, depletes ferrous iron and prevent iron-dependent lipid peroxidation	Wang et al., 2016
Cycliprox	Iron	Function as iron chelator, depletes iron and prevent iron-dependent lipid peroxidation	Stockwell et al., 2017
VK-28	Iron	Function as iron chelator, depletes iron and prevent iron-dependent lipid peroxidation	Li et al., 2017
Ceruloplasmin	Iron	Function as ferroxidase, oxidizes toxic ferrous iron to less toxic ferric iron	Liu et al., 2019
Ferrostatins-1	ROS	Aggravates ROS generation and blocks lipid peroxidation	Zilka et al., 2017
Liproxstatin-1	ROS	Function as lipophilic antioxidants and blocks lipid peroxidation	Zilka et al., 2017
GPX4	ROS	Blocks lipid peroxidation	Zhang Z. et al., 2018
Vitamin E	ROS	May inhibit lipoxygenases and blocks lipid peroxidation	Hinman et al., 2018
Vitamin C	ROS	Inhibits lipid peroxidation	Tang and Tang, 2019
Beta-carrotene	ROS	Blocks lipid peroxidation	Kose et al., 2019
N-Acetylcysteine	System Xc-	Promotes cysteine import and cause GSH synthesis	Karuppagounder et al., 2018
Dopamine	GPX4	Function as neurotransmitter, blocks GPX4 degradation and blocks lipid peroxidation	Tang and Tang, 2019
Selenium	GPX4, selenoproteins	Drives antioxidant GPX4 expression and blocks lipid peroxidation, increase abundance of selenoproteins.	Alim et al., 2019
Zileuton	5-LOX	Inhibits cytosolic ROS production as 5-LOX inhibitor and blocks lipid peroxidation.	Liu et al., 2015
Ferroptosis Suppressor Protein 1 (FSP1)	CoQ10	reduce CoQ10 to generate a lipophilic RTA that halts the propagation of lipid peroxides	Bersuker et al., 2019

### Inhibitors of Iron Metabolism

#### Iron Chelators

Because ferroptosis is characterized by the iron-induced accumulation of lipid ROS, iron is essential for the accumulation of lipid peroxides and the consequent execution of ferroptosis. As inhibitors of iron metabolism, iron chelators deplete iron by reducing the Fe^2+^ in LIP to prevent iron-dependent lipid peroxidation (Doll and Conrad, 2017), reduce the lipid ROS, and eventually inhibit ferroptosis. As a result, the potential therapeutic use of iron chelators has attracted substantial attention in recent years (De Domenico et al., 2009). Hu et al. showed that deferoxamine (DFO) treatment decreased hemin release from a hematoma, and demonstrated the potential role of DFO in reducing iron deposition and brain injury in a piglet model (Hu et al., 2019). However, a phase I clinical trial found that DFO led to hypotension, pancytopenia, retinal toxicity, and neurotoxicity (Selim et al., 2011). Li et al. (2017) further identified VK-28 as an iron chelator with similar effects to those of DFO in a rat model. However, compared with DFO, VK-28 improves neurobehavioral performance by polarizing the microglia to an M2-like phenotype, which reduces brain water content, decreases white matter injury, and eventually reduces the overall mortality due to ICH. Selim et al. (2019) showed that DFO was ineffective in improving the clinical outcome on day 90 in ICH patients of the phase II clinical trials. In this context, VK-28 appears to be more effective and safer than DFO. Another iron chelator, deferiprone (DFP), showed efficacy to some extent in humans and experimental neurodegenerative and neurodevelopmental conditions (Garringer et al., 2016; Carboni et al., 2017; Tian et al., 2017; Shao et al., 2020), but a phase II clinical study shows that it caused injury to the surrounding tissue (Klopstock et al., 2019). Therefore, the clinical application of DFO is limited. Wang et al. (2016) found that in rat models, DFP could reduce iron contents after ICH, but it was found to be ineffective against brain edema and lipid ROS and failed to improve the outcome. Furthermore, some studies found that minocycline functions as an iron chelator and can reduce iron-induced neuronal cell death (Zhao et al., 2011a; Chang et al., 2017; Fouda et al., 2017; Yang et al., 2019, 2020). Minocycline is being investigated in the phase II clinical trials of Zhao et al. (2011a), which shows that minocycline inhibited iron overload, reduced iron neurotoxicity and iron-induced brain injury after ICH, which can be regarded as a new treatment option of ICH. Yang et al. (2020) shows that minocycline reduced the brain edema and hematoma volume, prevented iron accumulation, and protected brain from iron-induced injury in ICH minipig models. Several other iron chelators, including nitrilotriacetic acid, ethylenediaminetetraacetic acid, and clioquinol, are effective for treating ICH, as demonstrated in animal models (Wang et al., 2016; Kose et al., 2019). Since iron chelators are mainstream drugs of ferroptosis after ICH, the outcomes of using iron chelators in clinical and preclinical studies are summarized in Table 3.

**TABLE 3:** Outcomes of some major iron chelators used in ICH in the clinical and preclinical studies.

Iron chelator	Type of trial	Development Phase	Outcomes	References
Deferoxamine	Preclinical trials	Preclinical	Decreased hemin release from the hematoma and reduced iron deposition and the severity of brain injury in animal models.	Hu et al., 2019
	Clinical trials	Phase I and II, under investigation	Effective to a certain extent, but lead to hypotension, pancytopenia, retinal toxicity, and neurotoxicity, and would be ineffective to significantly improve the good clinical outcome at day 90 in ICH patients.	Hu et al., 2019; Selim et al., 2019
Deferiprone	Preclinical trials	Preclinical	Reduced iron deposition, but invalid to brain edema and lipid ROS, and failed to improve the outcome in rat models.	Wang et al., 2016
	Clinical trials	Phase II	Effective to some extent in humans and experimental neurodegenerative and neurodevelopmental conditions, but can cause injury to the surrounding tissue.	Klopstock et al., 2019
Minocycline	Preclinical trials	Preclinical	Reduced the hematoma volume and brain edema, prevented iron accumulation, and protected brain from injury after ICH in rat models.	Zhao et al., 2011a; Yang et al., 2019
	Clinical trials	Phase II, under investigation	Reduced iron overload and iron-induced brain injury after ICH.	Chang et al., 2017; Fouda et al., 2017; Yang et al., 2020
VK-28	Preclinical trials	Preclinical	Improved neurobehavioral performance, reduced brain water content, decreased white matter injury.	Li et al., 2017
Clioquinol	Preclinical trials	Preclinical	Improved the neurological outcome, attenuated brain edema, and ROS production in rat models.	Wang et al., 2016
Ceruloplasmin	Preclinical trials	Preclinical	Reduced the severity of brain injury in rat models.	Liu et al., 2019

#### Ferroxidase

Ferroxidase is another type of iron metabolism inhibitor. Ferroxidase can oxidize toxic Fe^2+^ iron to the less toxic Fe^3+^ iron and reduce the Fe^2+^ in LIP, reduce lipid ROS, and inhibit ferroptosis. In a series of studies, Liu et al. (2019) showed that ceruloplasmin is an essential ferroxidase, with the potential to reduce the severity of brain injury due to ferroptosis after ICH in a rat model. Although numerous previous studies showed that ceruloplasmin deficiency is associated with Alzheimer’s disease, Parkinson’s disease, and other neurodegenerative diseases in patients and rat models (Jiang et al., 2015; Stelten et al., 2019; Diouf et al., 2020; Liu et al., 2020; Shang et al., 2020), little is known about the therapeutic function of ceruloplasmin in ferroptosis after ICH. Moreover, clinical studies on ferroxidase in ICH patients are limited. Thus, additional studies are required to identify the specific types and roles of ferroxidase.

### Promoters of GSH Biosynthesis

Glutamate and cysteine are important regulators of ferroptosis. As intracellular glutamate is pumped out of the cell and exchanged for extracellular cysteine by system Xc- to synthesize GSH and inhibit ferroptosis (Dixon et al., 2012, 2014); increasing cysteine concentration is a potential therapeutic strategy for ICH. *N*-acetylcysteine (NAC) is a cysteine prodrug that increases the accumulation of cysteine in neurons. Karuppagounder et al. (2018) showed that NAC has therapeutic effects as a precursor for GSH, which acts on system Xc- to promote cysteine transfer into neuronal cells and targets lipid-derived reactive electrophilic species resulting from increased arachidonate 5-lipoxygenase, along with GSH-dependent enzymes (e.g., GSH *S*-transferases). Simultaneously, exogenous infusion of a clinically approved protective lipid species, prostaglandin E2, reduces the NAC concentration required to stimulate protection and functional recovery *in vitro* and *in vivo* in mice (Zhao et al., 2015; Karuppagounder et al., 2018). Although nearly all of these studies used safe doses of NAC, the dose required to induce a therapeutic effect is unclear. Thus, further studies are required to determine the precise dose of NAC required for the efficient control of brain injury after ICH.

### Inhibitors of Lipid ROS Generation

Reactive oxygen species production is the immediate cause of ferroptosis after ICH; thus, inhibiting ROS production is important to prevent ferroptosis in neurons after ICH (Ursini and Maiorino, 2020). During lipid oxidation, GPX4 is the primary enzyme that prevents ferroptosis (Stockwell et al., 2017; Seibt et al., 2019). Many studies have shown that ROS inhibitors (e.g., ferrostatins-1, liproxstatin-1, vitamin E, vitamin C, and beta-carotene), along with GPX4 and its promoters (e.g., dopamine and selenium), are effective in preventing ferroptosis in animal models (Imai et al., 2017; Stockwell, 2018; Bai et al., 2019; Chen X. et al., 2019; Tang and Tang, 2019; Stockwell and Jiang, 2020).

For example, the ROS inhibitors ferrostatin-1 and vitamin C aggravate ROS generation and block lipid peroxidation, and vitamin E may inhibit lipoxygenases (Zilka et al., 2017; Hinman et al., 2018; Chen B. et al., 2019). Dopamine and selenium are GPX4 inducers. Dopamine functions as a neurotransmitter to block GPX4 degradation, whereas selenium drives the expression of GPX4 to increase the abundance of selenoproteins (Alim et al., 2019; Tang and Tang, 2019), and both GPX4 inducers eventually block lipid peroxidation. Moreover, inhibitors targeting 5-lipoxygenase (e.g., zileuton) inhibit AA from oxidizing to AA-OOH-PE, suppress cytosolic ROS production, and block lipid peroxidation (Liu et al., 2015). Several recent reports have also indicated that ferroptosis suppressor protein 1, a GSH-independent ferroptosis suppressor, is recruited to the plasma membrane, where it reduces coenzyme Q10 to produce lipophilic free radicals and capture antioxidants via oxidoreductase, thereby preventing the production of lipid peroxides (Bersuker et al., 2019; Doll et al., 2019; Chen and Xie, 2020; Hadian, 2020). Another regulator of potential therapeutic significance is nuclear factor erythroid 2-related factor 2 (Dodson et al., 2019; Anandhan et al., 2020; Song and Long, 2020), which regulates hundreds of genes, including several genes directly or indirectly involved in modulating ferroptosis. Therefore, nuclear factor erythroid 2-related factor 2 regulates not only lipid metabolism, but also GSH and iron metabolism, and continues its mitochondrial function (Abdalkader et al., 2018).

## Conclusion

**FIGURE 1:**
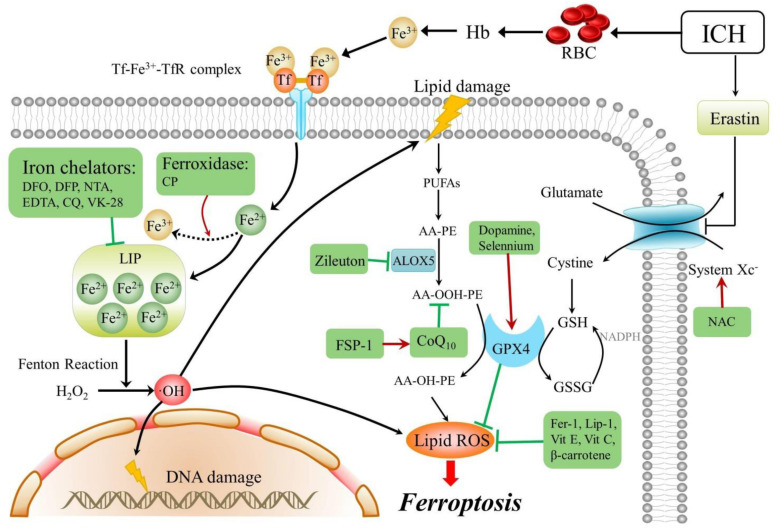
Mechanisms and modulators of ferroptosis after ICH. Arrows indicate promotion, blunt- ended lines indicate inhibition, and the drugs in a green box are ferroptosis inhibitors. ICH, intracerebral hemorrhage; RBC, red blood cell; Tf, transferrin; TfR, transferrin receptor; AA, arachidonic acid; PE, phosphatidylethanolamine; PUFAs, plasma membrane polyunsaturated fatty acids; CoQ 10, coenzyme Q10; DFO, deferoxamine; DFP, deferiprone; NTA, nitrilotriacetic acid; EDTA, ethylenediaminetetraacetic acid; CQ, clioquinol; CP, ceruloplasmin; H2O2, hydrogen peroxide; OH, hydroxyl radical; Fer-1, ferrostatin-1; GPX4, glutathione peroxidase 4; Vit E, vitamin E; Vit C, vitamin C; FSP-1, ferroptosis suppressor protein 1; GSH, glutathione; GSSG, oxidized glutathione; Hb, hemoglobin; Lip-1, liproxstatin-1; NAC, N-acetylcysteine; LOX, lipoxygenase; and ROS, reactive oxygen species.

**FIGURE 2:**
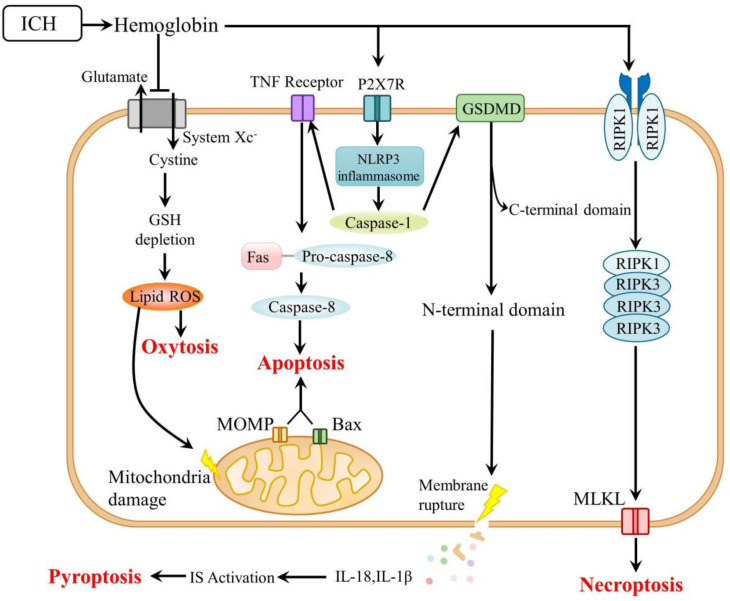
Mechanisms of other types of cell death after ICH. ICH, intracerebral hemorrhage; GSH, glutathione; ROS, reactive oxygen species; TNF, tumor necrosis factor; MOMP, mitochondrial outer membrane permeabilization; P2X7R, P2X7 receptor; NLRP3, nucleotide-binding oligomerization domain-like receptor 3; GSDMD, gasdermin D; RIPK, receptor-interacting protein kinase; MLKL, mixed lineage kinase domain-like; and IS, immune system.

The mechanisms underlying ferroptosis and its potential modulators after ICH are reviewed and are schematically summarized in Table 2 and Figure 1. The mechanisms of other types of programmed cell death are summarized in Figure 2. As a newly recognized form of programmed cell death, ferroptosis has attracted substantial attention for the development of new strategies to treat ICH and prevent SBI. Since 2012, rapid improvements have been made in elucidating the mechanism underlying ferroptosis and its regulation. Ferroptosis involves intracellular iron accumulation, GSH depletion, and lipid peroxidation. Thus, in theory, ferroptosis can be suppressed by iron chelators (e.g., DFO and DPF), lipophilic antioxidants (e.g., ferrostatin, liproxstatin, and vitamin E), and GSH biosynthesis promoters (e.g., NAC). Because iron plays a critical role in activating ferroptosis after ICH, iron chelators are regarded as mainstream drugs. However, several studies have indicated that DFO and DFP failed to improve the outcome of ICH, and the roles of iron and lipid autoxidation in ICH are not completely clear and should be further examined. Moreover, hematoma resolution and the clearance and phagocytosis of red blood cells might also reduce iron-induced ferroptosis, and they might therefore be considered potential therapeutic targets, warranting further clinical studies.

Currently, several molecules are known to regulate ferroptosis by directly or indirectly targeting iron metabolism, GSH biosynthesis, and lipid peroxidation. Some of these regulators (e.g., BID and NADPH) have also been implicated in other types of programmed cell death. For example, BID is involved in ferroptosis and oxytosis, and NADPH links ferroptosis and necroptosis. Moreover, these various forms of cell death are not independent but are rather intricately connected, forming a network to mediate cell damage after ICH. Therefore, ferroptosis is always accompanied by other types of programmed cell death. Accordingly, an important objective for further research on ferroptosis is to identify the signaling pathways, executors of iron-dependent ROS metabolism, and ultrastructural features of each type of programmed cell death. Thus, ferroptosis can be distinguished from the other types of cell death and improved therapeutic effects against each type of cell death can be achieved.

However, several issues remain to be addressed. First, it is essential to determine whether the regulation of ferroptosis is influenced by different types of brain cells, physiological conditions, individual lifestyles, and other factors. Second, more clinical trials should be conducted to test the effects of ferroptosis inhibitors, which represent the most promising strategy to prevent SBI after ICH. Thus, further studies and clinical trials are expected to improve the understanding of ferroptosis and aid the development of new strategies for effective treatment of ICH.

## Author Contributions

S-YZ and G-ZC searched the literature and drafted the manuscript. X-LY, XW, YQ, Z-NG, and HJ critically revised the manuscript. All authors have made contributions to the work and approved it for publication.

## Conflict of Interest

The authors declare that the research was conducted in the absence of any commercial or financial relationships that could be construed as a potential conflict of interest.
